# Editorial: Methods for Single-Cell and Microbiome Sequencing Data

**DOI:** 10.3389/fgene.2022.920191

**Published:** 2022-05-13

**Authors:** Himel Mallick, Lingling An, Mengjie Chen, Pei Wang, Ni Zhao

**Affiliations:** ^1^ Biostatistics and Research Decision Sciences, Merck & Co.Inc., Rahway, NJ, United States; ^2^ Interdisciplinary Program in Statistics and Data Science, The University of Arizona, Tucson, AZ, United States; ^3^ Department of Epidemiology and Biostatistics, The University of Arizona, Tucson, AZ, United States; ^4^ Department of Biosystems Engineering, The University of Arizona, Tucson, AZ, United States; ^5^ Department of Human Genetics and Department of Medicine, University of Chicago, Chicago, IL, United States; ^6^ Tisch Cancer Institute, Icahn School of Medicine at Mount Sinai, New York, NY, United States; ^7^ Department of Genetics and Genomic Sciences, Icahn School of Medicine at Mount Sinai, New York, NY, United States; ^8^ Department of Biostatistics, Johns Hopkins University Bloomberg School of Public Health, Baltimore, MD, United States

**Keywords:** microbiome, single-cell, omics, data science, multi-omics, statistics, biostatistics, computational biology

Translational investigations of single-cell transcriptomics and microbiomics now constitute the research hotspots in the field of omics sciences with cell-type-specific gene expression and host-associated microbes and microbial gene products implicated in numerous complex diseases (Mallick et al., 2017; Aldridge and Teichmann, 2020). Motivated by the structural similarities of scRNAseq and metagenomics data (Calgaro et al., 2020; Jeganathan and Holmes, 2021), with respect to several statistical properties such as, high-dimensionality, count and compositional nature, excess zeros due to low sequencing depth or dropout, overdispersion, and spatial and temporal dependence, among others, we set out to launch a combined Research Topic following the completion of the successful first volume (Mallick et al., 2020) in 2020.

**FIGURE 1:**
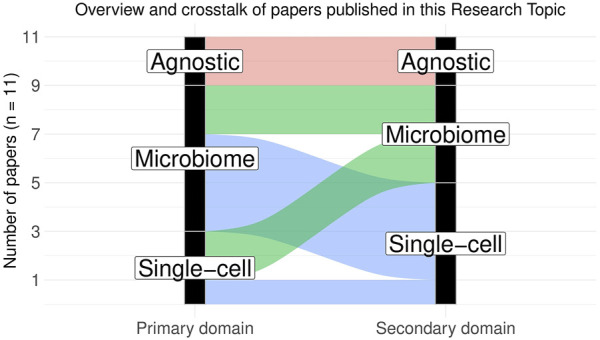
Overview and crosstalk of papers published in this Research Topic. The papers can be broadly categorized into four subtypes: 1) domain-agnostic publications generally applicable to both microbiome and single-cell studies (2 papers), 2) single-cell-specific methods that can be potentially applied to microbiome data with necessary modifications (2 papers), 3) microbiome-specific methods that can be potentially applied to scRNASeq data with necessary modifications (4 papers) and 4) domain-specific analysis methods or literature reviews (1 single-cell-specific and 2 microbiome-specific papers). Primary domain indicates the intended application area for the related paper, whereas secondary domain indicates the potential application area wherein the said method can be extended with necessary modifications.

This Research Topic thus consists of eleven papers (including the editorial) on various single-cell and microbiome omics areas and covers the latest development of statistical methods for analyzing microbiome and single-cell sequencing data. The papers can be broadly categorized into four subtypes (Figure 1): 1) Specialized domain-specific publications, 2) domain-agnostic publications applicable to both microbiome and single-cell studies, 3) single-cell-specific methods with potential applicability to microbiome studies, and 4) microbiome-specific methods with potential applicability to scRNASeq.

One of the most common applications of omics data is the differential expression or abundance analysis to identify omics features that are differential between two or more biological conditions. Despite being a well-studied problem, differential analysis is still a very active area of research. In both single-cell and microbiome studies, given the large number of features present in a typical dataset, standard statistical testing procedures can put false association or loss of power at odds with prior knowledge or expectations (Mallick et al., 2017). While most of the current methods are domain- or platform-specific, domain-agnostic methods applicable to multiple platforms or data types are becoming increasingly common (Mallick et al., 2021a; Rahnavard et al., 2021). Taking advantage of the inherent compositionality and hierarchical tree structure observed in both single-cell and microbiome sequencing data, Ostner et al. proposes a domain-agnostic Bayesian tree-aggregated model (tascCODA) applicable to any compositional rectangular data with hierarchical row or column information. tascCODA thus constitutes a valuable addition to the growing statistical toolbox of domain-agnostic methods for omics research enhancing interoperability of disparate omics datasets (Sansone et al., 2009; Conesa and Beck, 2019).

A popular alternative to per-feature differential abundance analysis methods is the community-level or omnibus association methods that enable associating the entire microbial community composition with a phenotype of interest (Mallick et al., 2017). Due to their multivariate setups, omnibus association methods typically fail to provide feature-level inference to enable follow-up characterization (Mallick et al., 2021b). To this end, Chen et al. proposes a hybrid method (MiAF) that adaptively combines *p*-values from the feature-level tests to construct a community-level test, thus providing the best of both worlds in a unified framework. Jiang et al. extends the popular community-level test (MiRKAT) to multi-categorical nominal and ordinal outcomes for both independent or clustered (e.g., family-based and longitudinal) microbiome studies.

Keeping pace with ongoing advances in artificial intelligence, a variety of machine learning methods have become available to analyze microbiome and single-cell data. Deek and Li proposes a Bayesian data generative process for microbiome community data by developing a zero-inflated Latent Dirichlet Allocation (zinLDA) model that accurately identifies the latent sparse subcommunities of a microbial community, improving upon the state-of-the-art Latent Dirichlet Allocation (LDA) model. Zhang et al. develops a novel, unsupervised, data-driven deep learning-based imputation method (NISC) to impute the excess amount of zeroes (dropouts) observed in scRNA-seq count data that improves downstream cell type identification accuracy compared to existing imputation methods.

Just as differential analysis provides one potential area to transfer methods between fields, inference of feature-feature interaction network estimation provides another. Improving upon the existing cross-sectional ecological network inference methods, He et al. proposes a novel autoregressive zero-inflated Poisson mixed-effects model (ARZIMM) to detect sparse microbial interactions in longitudinal microbiome data, thus providing a scalable alternative to existing computationally intensive temporal ecological network detection and stability estimation methods.

Both microbial community and single-cell datasets possess unique characteristics that differ in ways that necessitate the development of domain-specific tools, with many of the single-omics tools not susceptible to technological variability induced by experimental platforms or library preparation protocols (Mallick et al., 2021a). To this end, several domain- and platform-specific methods and literature reviews have been published to better address the biological question at hand within a specific context.

Wu et al. proposes a non-linear normalization approach for non-UMI single-cell data that reduces more technical variation than competing methods without reducing biological variation. Jones et al. asserts that in 16S rRNA gene sequencing data (specially in the Ion Torrent platform), assessing multiple hypervariable regions in tandem is critical to enhance the statistical evaluation of overall differences in community structure and relatedness among samples. Paisley and Liu develops and deploys an R Shiny web tool (GeneMarkeR) in order to provide a vastly expanded, standardized marker gene database for the end users, improving upon existing overwhelmingly incoherent databases often with a lack of validated standards. Finally, Arbas et al. carefully curates the literature to highlight the current state-of-the-field in longitudinal microbiome studies ranging from experimental design and basic bioinformatics preprocessing steps to critical multi-omic data integration considerations including modeling, validation, and inference.

Many of the methods described in this Research Topic also come with accompanying open-source software implementations, thus providing an important resource for future methodologists and machine learners and many of them are potentially extensible to other data types beyond their intended application domains (Figure 1). As the field of omics research progresses, we expect to see more research linking disparate omics data with human genetics and digital pathology in order to gain better functional insights into the role of omics features in disease initiation and progression. We also expect to see more diverse data sets at the intersection of spatial omics, long-read sequencing, and imaging genomics, giving rise to new statistical questions and challenges, which motivated us to launch a third volume of the Research Topic on imaging and omics data science. We hope that omics and imaging scientists from various subfields will work together in this exciting area of research and make important scientific contributions by providing a shared infrastructure for common data types and fostering ideas for more sophisticated, reproducible, interpretable data analyses.
